# Red maple tree root water uptake depths are influenced by neighboring tree species composition

**DOI:** 10.1093/treephys/tpaf049

**Published:** 2025-04-23

**Authors:** Matthew Sobota, Kevin Li, James Knighton

**Affiliations:** Department of Natural Resources and the Environment, University of Connecticut, 1376 Storrs Rd. Storrs, CT 06268, USA; Department of Natural Resources and the Environment, University of Connecticut, 1376 Storrs Rd. Storrs, CT 06268, USA; Department of Natural Resources and the Environment, University of Connecticut, 1376 Storrs Rd. Storrs, CT 06268, USA

**Keywords:** drought, ecohydrological modeling, mixed-species forests, rooting depth, stable isotopes in water, water competition

## Abstract

Understanding how mixed-species forests uptake subsurface water sources is critical to projecting future forest water use and stress. Variation in root water uptake (RWU) depths and volumes is common among trees but it is unclear how it is affected by species identity, local water availability or neighboring tree species compositions. We evaluated the hypothesis that RWU depths and the age of water (i.e., time since water entered soils as precipitation) taken up by red maples (*Acer rubrum*) varied significantly between two forested plots, both containing red maples, similar soils, topography and hydrologic conditions, but having different neighboring tree species. We measured soil moisture contents as well as stable isotopes (δ^2^H, δ^18^O) in plant xylem water and soil moisture across two years. These data were used to calibrate process-based stand-level ecohydrological models for each plot to estimate species-level RWU depths. Model calibration suggested significant differences in red maple tree RWU depths, transpiration rates and the ages of water taken up by maples across the two stands. Maple trees growing with ash and white spruce relied on significantly deeper and older water from the soil profile than maple trees growing with birch and oak. The drought risk profile experienced by maple trees differed between the plots as demonstrated by strong correlations between precipitation and model simulated transpiration on a weekly time scale for maples taking up shallow soil moisture and a monthly time scale for maples reliant on deeper soil moisture. These findings carry significant implications for our understanding of water competition in mixed-species forests and for the representation of forest rooting strategies in hydrologic and earth systems models.

## Introduction

Root water uptake (RWU) and the transpiration of water by plants drive primary productivity and are a substantial component of the hydrologic cycle (Jasechko et al. 2014, Good et al. 2015, Makarieva et al. 2023). Understanding how RWU occurs in mixed-species stands is critical to forecasting forest productivity, health and water yields under shifting climatic and species composition change (Ford et al. 2011*b*, González de Andrés et al. 2018). Variations in RWU depths support the ability of forests to maintain stable transpiration and landscape water runoff. Root water uptake can play a strong role in both forest resistance (i.e., the magnitude of plant function change after disturbance) and resilience (i.e., the rate of recovery following disturbance) to external perturbations (e.g., drought, fire, pest infestations, disease) (Jactel and Brockerhoff 2007, Cardinale et al. 2011, Forrester et al. 2017, Georgi et al. 2021). Resolving forest rooting patterns is critical for developing accurate forecasts of water and carbon cycling in regional hydrologic and earth systems models (Fisher et al. 2018, Gao et al. 2024, Jimenez-Rodriguez et al. 2024).

Mixed-species forested stands often contain trees with different maximum rooting depths (Gale and Grigal 1987, Mueller et al. 2013) and RWU from isotopically distinct sources of belowground water (Allen et al. 2019, Knighton et al. 2020*b*, Nehemy et al. 2022, Floriancic et al. 2024), though clear explanations for these variations are lacking. Differences in maximum rooting depths have been posited as a primary determinant of tree vulnerability to drought (Ivanov et al. 2012, Chitra-Tarak et al. 2018, 2021, Ye et al. 2019, Kahmen et al. 2022). Neighboring trees compete for a potentially limited source of water when they have similar water uptake depths (Gaines et al. 2016, Lechuga et al. 2017, Magh et al. 2020). Trees can also engage in positive interactions, such as facilitative hydraulic redistribution of water sources from wetter to drier soil layers (Hafner et al. 2017). Higher taxonomic diversity may therefore imply higher diversity of water acquisition strategies in mixed-species forests and possibly increased drought resistance (Anderegg et al. 2016, Forrester and Bauhus 2016, Grossiord 2020, Vannoppen et al. 2020, Haberstroh and Werner 2022). However, substantial prior observed variations in productivity and resilience exhibited by mixed-species forests (Grossiord et al. 2014, Forrester and Bauhus 2016, Pardos et al. 2021, Knighton and Berghuijs 2023, Mas et al. 2024) may not be attributable to taxonomic diversity.

Several conceptual models aim to explain variation in forest stand rooting patterns. One simple model posits that plants employ rooting strategies that depend solely on species identity. Phylogenetic analysis of global maximum rooting depths suggests that about half of global variation in rooting depth can be explained by phylogenetic relatedness (Knighton et al. 2024). An alternative conceptual model suggests that all tree rooting and water uptake depths are defined by the local mean water table depth (Fan et al. 2017, 2019); however, this conceptual model does not explain why strong phylogenetic signals are present in rooting depths (McCormack et al. 2020, Knighton et al. 2021, Ávila-Lovera et al. 2023), why species have been observed to compete for shallow soil moisture despite available water in deeper soils or groundwater (Gaines et al. 2016, Lechuga et al. 2017, Magh et al. 2020), or cases where neighboring tree species take up different sources of water (Allen et al. 2019, Bello et al. 2019, Knighton et al. 2020*b*, Nehemy et al. 2022, Floriancic et al. 2024).

A third possibility is that plant RWU depths are influenced by competition for below ground water sources among neighboring tree species within mixed-species stands. There is strong empirical evidence that species identity, neighboring species compositions and local water availability determine plant water-use strategies (Bhuyan et al. 2017, Vitali et al. 2018, Vitasse et al. 2019, Harley et al. 2020, Gutierrez Lopez et al. 2021, Liu et al. 2021, Schoppach et al. 2021, Fresne et al. 2023, Charlet de Sauvage et al. 2024). In a dense tropical ecosystem with high species diversity, rooting strategies were a primary axis defining productivity (Brum et al. 2019), suggesting niche partitioning (i.e., dividing resource use by species to avoid competition) of subsurface water. Case studies have demonstrated that the presence of roots of neighboring trees can spur both vertical and lateral root growth across stands (Cabal et al. 2020, Agee et al. 2021), which may influence RWU depths.

The goal of this project was to determine whether RWU depths varied between stands with differing tree species compositions. We first tested the hypothesis that the RWU depth of red maple trees (*Acer rubrum*) in paired mixed-species plots with different neighboring tree species was significantly different despite existing in the same climate with similar soils and root zone water availability. We then tested the hypothesis that variations in rooting strategies employed by maple altered the age and volume of water transpired by both maples and neighboring tree species.

## Materials and methods

### Field site description and stand-level tree characteristics

**Figure 1:**
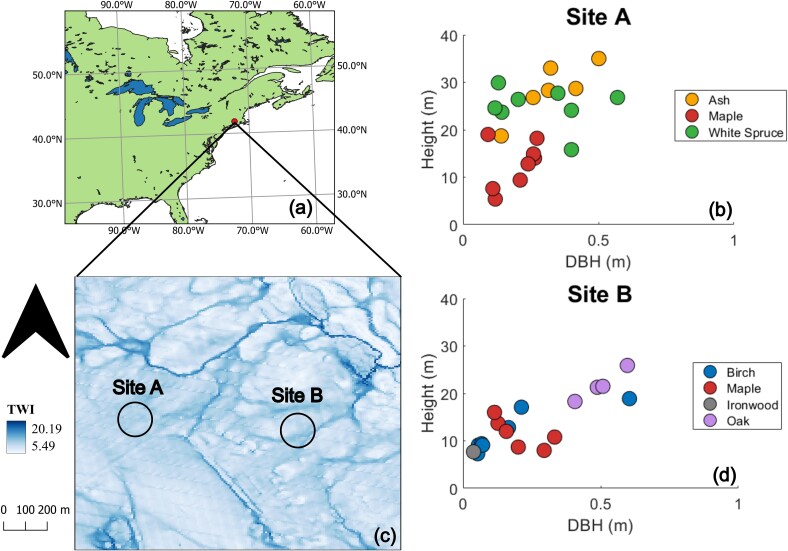
(a) Field site location on a map of the eastern USA and Canada, (b) and (d) stand-level tree characteristics for the surveyed subplots within Sites A and B, and (c) 10 m Topographic Wetness Index (TWI) of land parcel containing Sites A and B.

This study was conducted in paired 900 m^2^ mixed-species forested plots (hereafter referred to as Sites A and B) within the University of Connecticut Forest (41.82°, −72.25°) (Figure 1a and c). The regional mean annual precipitation is 1410 mm^1^year^−1^ and mean annual temperature is 9.7 °C. Across the historical meteorological record, the frost-free duration ranged from 140 to 240 days (NCDC 2024). Surface slopes varied from 0 to 8% across Site A and 0 to 15% across Site B. The distribution of Topographic Wetness Indices (TWI) (Fairfield and Leymarie 1991) derived from 10 m DEM (USGS 2023) in SAGA using the Rho8 method were similar across the two plots (Site A median = 9.53, Site B median = 8.74), suggesting similar hydrologic conditions (Figure 1c). Root zone soils at both sites were Woodbridge series fine sandy loam with a high gravel content and reported available water supplies of 119 mm and 102 mm at Sites A and B, respectively (NRCS 2019). From June through October 2022, the region experienced a substantial drought defined via the US Drought Monitor drought classification for northeastern CT (USDM 2024), characterized by low precipitation totals, high daily mean temperatures and high vapor pressure deficit (VPD) (Jones 1992) (Figure S1 available as Supplementary data at *Tree Physiology* Online). The durations of drought categories ranging from D1 (moderate) to D3 (extreme) were determined as the first day and last day reported for the Shetucket watershed (USDM 2024).

Site A regenerated to forest from pasture beginning ~1950. Spruce trees (*Picea glauca*) were introduced whereas ash (*Fraxinus* sp.) and maple (*A. rubrum*) naturally established. Site B naturally regenerated to forest from pasture beginning ~1910, resulting in a mixture of ash, birch (*Betula* sp.), oak (*Quercus alba* and *Quercus rubra*) and maple. Spruce and ash trees were thinned at Site A in 2013 and ash trees completely salvaged from Site B in 2016 due to concern of potential future emerald ash borer infestation. Tree surveys were conducted at both sites in October 2021 and again in June 2023 to establish tree species composition, density and size structure. Three 12 m diameter subplots were randomly selected within each site. Within each subplot, tree diameter at breast height (DBH) was measured with DBH tapes (Forestry Suppliers Inc. Diameter Tape Model 283D/20F). Tree heights were measured with ground tapes (Crescent Lufkin Long Tape Measure Model FM030DM) and clinometers (Suunto Model PM-5/360 PC). Site A was composed of ash, maple and white spruce. The basal areas of ash, maple and white spruce in Site A were 14.8, 27.8 and 34.1 m^2^ hectare^−1^, respectively. Basal areas of birch, maple and oak in Site B were 15.2, 29.7 and 24.4 m^2^ hectare^−1^, respectively. The upper canopy of Site A was occupied by ash trees, generally positioned above both the maple and white spruce canopies (Figure 1b). In Site B, oak occupied the highest canopy position, above both birch and maple (Figure 1d).

Estimates of the root mass distribution of each plot was determined by collecting 200 g samples of soil and roots at depths of 10, 20, 30, 40, 50, 75 and 100 cm depth across three randomly selected soil coring locations in each plot. The maximum depth of samples was determined by high soil-rock content limiting auger progress. Samples were oven dried at 105 °C and sieved for roots. Dried soils and roots were weighed to determine the dry weight of fine roots at each depth. Root densities measured in both sites were approximately uniformly distributed between the surface and 0.5 m depth, with some fine roots found at 1 m depth in both sites (Figure S2 available as Supplementary data at *Tree Physiology* Online).

### Field sample collection and analysis

Soil moisture content measurements, soil samples and plant xylem samples were collected monthly within each plot from June through August in 2021 and 2022. Shallow soil (top 12 cm) volumetric water content (VWC) was measured at five randomly selected locations within each plot with a HydroSense II Handheld Soil Moisture Sensor. Soil and tree core samples were collected for water isotopic analysis (^2^H and ^18^O). Approximately 150 g of soil was collected at 5, 10, 20, 30, 40 and 50 cm depths in triplicate with an auger at a monthly interval. Concurrently, tree cores were collected from three randomly selected individuals of each of the most common tree genera within each site with a 5.15 mm diameter increment borer at ~1.5 m. The same individual trees were flagged and assigned a unique sampling identity at the start of sampling, and then sampled repeatedly on each sampling date. Samples were collected out of vertical alignment from all prior cored locations on the trunk. Collected soils and tree cores were stored in double-seal Ziploc brand bags and frozen within 2 h of collection. To evaluate the potential for evaporative fractionation from this sample transport methodology (Millar et al. 2022), we placed 50 mL of a liquid water standard into six double seal ziploc bags and stored them in the field collection bag in an indoor lab space. After 2 h had elapsed we sampled the water in the bags and observed a mean enrichment of +0.274‰ and +0.002‰ for δ^2^H and δ^18^O. Precipitation, wind speed, solar radiation and relative humidity were recorded at a daily interval ~1 km from the study location (Knighton 2024). Precipitation samples have been collected for isotopic analysis at a daily interval (when present) in a glass container with a funnel at a site 3.5 km away from the study sites since October 2020.

Bark and phloem were removed from xylem tissue for each tree core sample prior to extraction. Water was extracted from xylem via a Cryogenic Vacuum Extraction (CVE) system (Sobota et al. 2024). Samples were placed in glass vials and submerged in water with a minimum temperature of 80 °C. Collection vials were partially submerged in LN_2_ (−196 °C). The CVE was run with a vacuum pump with a pressure rating of 200 Pascals (Vaccubrand MD1C) for a minimum of 60 min. Samples with less than 97.5% water recovery were discarded from analysis. Further details on sample preparation and the CVE procedure are available in Sobota et al. (2024).

Liquid water samples (precipitation and extracted xylem water) were analyzed on a Picarro L-2130i with three water standards ranging from −98.68‰ to −18.70‰ for δ^2^H and −15.23‰ to −1.00‰ for δ^18^O. All samples were analyzed in high-throughput mode (~6 min of continuous analysis per sample) with six injections. We discarded the first three injections for each sample. Reported isotopic values were the average of the final three injections. All samples were screened for organic contamination (CH_4_ and alcohols) using the ChemCorrect software. Liquid water samples that were flagged for organic contamination were excluded from further analysis.

Soil moisture samples were analyzed for δ^2^H and δ^18^O via direct vapor equilibration (DVE) (Wassenaar et al. 2008). Soil samples were placed into aluminum-lined mylar bags. Bag head spaces were flushed with ultra dry air (10 p.p.m. H_2_O) and left to equilibrate for 48 h in a temperature-controlled room where the DVE analysis was performed. The same procedure was used for three bags filled with 10 mL of each of the liquid water standards (described above). After equilibration, the vapor in the headspace of each bag was sampled directly with a needle connected to a Picarro L-2130i. Water vapor concentrations and δ^18^O and δ^2^H were measured continuously for at least 6 min per sample. We used a rolling average to identify the most stable water vapor signals for each sample during analysis, which were then used to compute average δ^18^O and δ^2^H sample values. A linear calibration relationship was established for each sampling day and applied to all soil moisture δ^18^O and δ^2^H values by fitting a regression through measured and known water standard isotopic compositions.

### Ecohydrological model description and development

We developed ecohydrological models (EcH_2_O-iso) for each experimental site to produce process-based estimates of RWU depths, transpiration rates and water ages (Maneta and Silverman 2013, Kuppel et al. 2018*a*). EcH_2_O-iso is a process-based ecohydrological model capable of simulating plot-scale water and energy fluxes as well as plant growth, death and decay dynamics, stable water isotopic tracers in precipitation, soils, groundwater and plant xylem water, and water ages (Maneta and Silverman 2013, Kuppel et al. 2020). Prior research has demonstrated that calibration of EcH_2_O-iso to biophysical datasets can yield reliable parameterizations of soil physical properties (Maneta and Silverman 2013, Kuppel et al. 2018*a*) and plant economic and hydraulic traits (Li et al. 2023*a*). Though both δ^2^H and δ^18^O were measured in soil moisture and plant xylem water (described in ‘Field sample collection and analysis’ section) we simulated only δ^18^O due to potential for xylem water δ^2^H biases resulting from CVE (Chen et al. 2020, Wen et al. 2022, Sobota et al. 2024).

Soil textures and physical properties are homogenous across the upper 1.7 m (NRCS 2025). We therefore assume that calibration of a process-based soil water transport model to shallow soil VWC measurements and soil moisture isotopic observations across the upper 0.5 m provide a realistic calibration of soil water transport*.* Soils were simulated as three vertically stacked layers (L1, L2 and L3) which were discretized as 0–0.2 m, 0.2–0.4 m and 0.4–1.5 m. These soil layer depths were selected to capture significant vertical stratification of soil moisture δ^18^O in June and September 2021, and May through August 2022 (Figure S3 available as Supplementary data at *Tree Physiology* Online). Although the majority of observed root density was within the upper 0.5 m of the soil depth profile (Figure S2 available as Supplementary data at *Tree Physiology* Online), our model allowed for the possibility of deeper RWU. Rainfall and snowmelt were partitioned into surface runoff and infiltration at the surface based on the infiltration rate of soil layer L1 determined by saturated hydraulic conductivity, *KH*_SAT_ and the soil water content, $\theta$. The relationship between time-varying soil water contents and water potentials, *Ѱ*, were determined via the parameters soil porosity, *ϕ*, the residual volumetric soil water content, $\theta$_r_ and the Brooks–Corey *λ*_BC_ and *Ѱ*_ae_ (Eq. 1).


(1) \begin{equation*} \theta ={\left(\frac{\psi_{\mathrm{ae}}}{\psi}\right)}^{\lambda_{\mathrm{BC}}}\left(\phi -{\theta}_{\mathrm{r}}\right)+{\theta}_{\mathrm{r}} \end{equation*}

Percolate leaked from overlying layers with the mean isotopic composition and water age of the overlying soil layer. Groundwater outflow from the bottom of the soil profile was simulated as a function of water potential head and horizontal hydraulic conductivity. Rising groundwater, when present, is exchanged isotopically with each soil layer.

Evaporation (*E*) and plant transpiration (*T*) were modeled separately, where *E* occurs only from soil L1 and is simulated as a fractionating process whereas *T* is simulated as non-fractionating and can draw on all soil layers (Eq. 1). In both Sites A and B, the three focal tree clades (Site A: ash (*Fraxinus* sp.), maple (*A. rubrum*) and white spruce (*P. glauca*); Site B: birch (*Betula* sp.), maple (*A. rubrum*) and oak (*Quercus* sp.)) were simulated as co-occurring in a 1D stand model with their measured basal areas. For Site B, we grouped all white and red oaks into one plant type due to the rare occurrence of red oaks and similarity in reported hydraulic trait values between these species (Fraser 2020, Knighton 2024).

Each tree clade was defined by a set of plant parameters reflecting plant traits. The fractions of water uptake demand exerted on each of the three soil layers, ${f}_{\mathrm{root}}^{\mathrm{L}1,2,3}$, are defined via the root profile shape parameter, *K*_ROOT_, the total soil depth, *D*_soil_ and the depths of the first and second soil layers, *D*_L1_ and *D*_L2_ respectively (Eqs 2–4) (Kuppel et al. 2018*b*). The isotopic composition of tree RWU was the volumetric weighted composition of the isotopic contribution of each soil layer.


(2) \begin{equation*} {f}_{\mathrm{root}}^{\mathrm{L}1}=\frac{1-\exp \left({k}_{\mathrm{root}}\times{D}_{\mathrm{L}1}\right)}{1-\exp \left({k}_{\mathrm{root}}\times{D}_{\mathrm{soil}}\right)} \end{equation*}

(3) \begin{equation*} {f}_{\mathrm{root}}^{\mathrm{L}2}=\frac{\exp \left({k}_{\mathrm{root}}\times{D}_{\mathrm{L}1}\right)-\exp \left({k}_{\mathrm{root}}\times \left({D}_{\mathrm{L}1}+{D}_{\mathrm{L}2}\right)\right)}{1-\exp \left({k}_{\mathrm{root}}\times{D}_{\mathrm{soil}}\right)} \end{equation*}

(4) \begin{equation*} {f}_{\mathrm{root}}^{\mathrm{L}3}=1-{f}_{\mathrm{root}}^{\mathrm{L}1}-{f}_{\mathrm{root}}^{\mathrm{L}2} \end{equation*}

Canopy light interception was simulated via the Beers–Lambert relationship through the light attenuation coefficient parameter (*K*_BEERS_). Plant transpiration is determined by maximum stomatal conductance, *gs*_MAX_, the soil moisture contents of all three soil layers and the critical soil water tension at which stomatal conductance is 0, *Ѱ*_D_, leaf area index (LAI; where LAI is solved dynamically for each species based on leaf carbon allocation), atmospheric water demand and stomatal closure in response to high VPDs, where total conductance is reduced by a factor, ${f}_{\mathrm{ea}}$, based on a calibrated coefficient, *gsvpd* and the VPD.


(5) \begin{equation*} {f}_{\mathrm{ea}}\left(\mathrm{VPD}\right)=\exp \left[-gs vpd\left(\mathrm{VPD}\right)\right] \end{equation*}

### EcH_2_O-iso model calibration

Our model calibration modified the values of 33 parameters to best fit the model to the observed data that we collected in Sites A and B. These 33 parameters included 9 parameters describing soil water tensions and surface energy dynamics (Table S1 available as Supplementary data at *Tree Physiology* Online), and 8 parameters describing the water and energy use of each of the three simulated tree clades (Table S2 available as Supplementary data at *Tree Physiology* Online). The parameters selected for calibration and their prior ranges were based on prior sensitivity analyses using EcH_2_O-iso (Kuppel et al. 2018*b*, Douinot et al. 2019, Knighton et al. 2020*b*, Smith et al. 2020, Li et al. 2023*a*). The remaining parameters included in EcH_2_O-iso were determined to have low-sensitivity (Smith et al. 2020, Li et al. 2023*a*) and were left at their default values. We performed 30,000 simulations, uniformly randomly sampling each sensitive parameter based on feasible ranges defined in prior research (Tables S1 and S2 available as Supplementary data at *Tree Physiology* Online) (Maneta and Silverman 2013, Kuppel et al. 2018*b*, 2018*c*, Knighton et al. 2020*b*, Li et al. 2023*a*). For each simulation, we computed the root mean square error (RMSE) between shallow soil VWC, the isotopic composition of each soil layer (δ^18^O L1, δ^18^O L2 and δ^18^O L3), and the isotopic compositions of xylem water for each simulated tree clades (δ^18^O Ash, δ^18^O Maple and δ^18^O White Spruce in Site A and δ^18^O Birch, δ^18^O Maple and δ^18^O Oak in Site B). The observed values used for VWC, soil and xylem δ^18^O were the median of all measurements or samples collected on that sampling date. We defined the accepted model simulations and their associated parameters using the cumulative distribution function (CDF) goodness of fit (GOF) approach (Ala-aho et al. 2017). We simultaneously fit the model to soil VWC, soil δ^18^O across all layers and xylem water δ^18^O for all clades in each site. We conservatively accepted the 100 most representative simulations to define the median simulated time series of each state variable, uncertainty bounds and model parameter values. The calibrated models yielded probability distributions for model parameters, including clade-level *K*_ROOT_ parameters defining RWU depths for each tree clade in each plot.

### Statistical analyses

All statistics were computed using MATLAB ver. R2024b. We tested for significant differences in the calibrated RWU depth (*K*_ROOT_) distributions between all trees within each plot using two-sided Mann–Whitney *U*-tests (HA: the median calibrated *K*_ROOT_ values for two clades are different; H0: the median *K*_ROOT_ values are equal). For this and all subsequent tests, we assessed the significance at the *a* thresholds of 0.1, 0.05 and 0.01. We then repeated this test for significant differences between calibrated *K*_ROOT_ values between maple trees in Sites A and B.

We tested for significant differences between the median maple transpiration rates, evapotranspiration water ages and non-maple tree transpiration rates between Sites A and B during the 2022 drought (June–August) using Mann–Whitney *U*-tests. These tests were repeated three times using the D1, D2 and D3 drought classification boundaries to understand how drought duration influenced these results.

Finally, we aimed to determine how strongly correlated maple evapotranspiration and precipitation were across the two stands. High correlations between transpiration and precipitation for a period of 14 days or less suggests a strong reliance on recent precipitation whereas low correlations indicate that maple trees transpired a water source that was not rapidly replenished by rainfall (i.e., deeper soil moisture or groundwater). We computed the wavelet coherence between daily precipitation and maple transpiration across the study period for Sites A and B. We then computed the difference in the wavelet coherences to highlight significant differences in the dependence on summer precipitation between maples in Sites A and B.

## Results

### Impact of neighboring species on maple root water uptake depths

**Figure 2:**
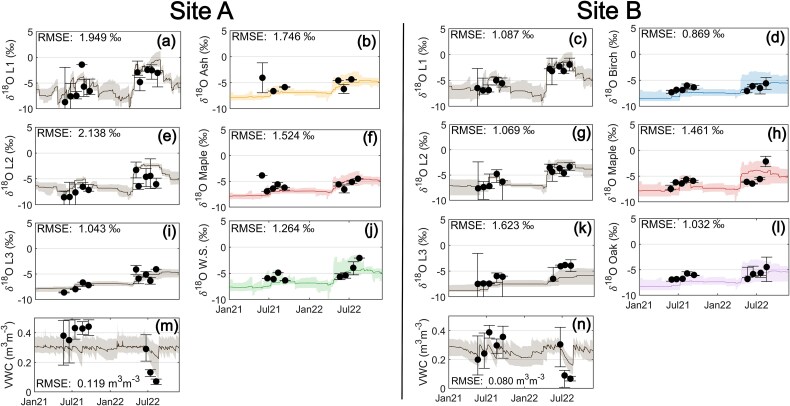
EcH_2_O-iso model calibration of dynamics in (m) and (n) shallow soil volumetric water content (VWC), (a, e, i); (c, g, k) soil water δ^18^O at three depth layers (L1, L2 and L3), and (b, f, j); (d, h, l) xylem water δ^18^O in each tree species across the study period at Site A and Site B. All simulated water dynamics show 95% uncertainty bounds. Black points represent the median observed measurements and error bars show the minimum and maximum observed measurements collected on that sampling date. Root mean square error (RMSE) was computed between observed and simulated shallow soil VWC, the isotopic composition of each soil layer (δ^18^O L1, δ^18^O L2 and δ^18^O L3), and the isotopic composition of xylem water for each tree species (δ^18^O Ash, δ^18^O Maple and δ^18^O White Spruce in Site A and iδ^18^O Birch, δ^18^O Maple and δ^18^O Oak in Site B).

EcH_2_O-iso models for Sites A and B reproduced the dynamics of soil moisture, soil water δ^18^O across all three rooting zone soil layers, and xylem water δ^18^O of all three clades in each plot (Figure 2). The quality of the model fit to observations is similar to other studies employing multi-objective model calibration to critical zone measurements (Kuppel et al. 2018*a*, Douinot et al. 2019, Knighton et al. 2020*a*, Smith et al. 2022, Li et al. 2023*b*, Wu et al. 2025). The median CDF simulation slightly overestimated the soil moisture content during the peak of the 2022 drought at both sites, potentially due to overestimation of summer precipitation at the long-term meteorological site. The isotopic compositions of ash and white spruce were underestimated for one sample each which may be attributable to a xylem water δ^18^O measurement bias arising from imperfect sample storage, transport, extraction or analysis (Millar et al. 2022, von Freyberg et al. 2022, Duvert et al. 2024), or the sampling strategy not fully capturing the natural isotopic heterogeneity within the stand (Li and Knighton 2023). Cumulative distribution functions for all calibrated soil and plant parameters are presented in Figures S4–S7 available as Supplementary data at *Tree Physiology* Online. All uncertainty in the simulated water fluxes and estimated parameter values are carried into subsequent hypothesis tests.

**Figure 3:**
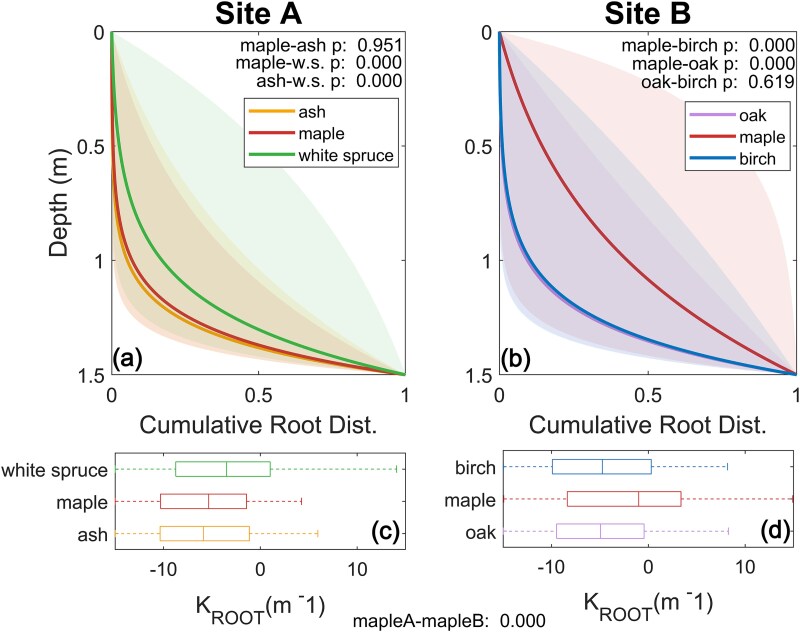
(a, b) Simulated rooting depths distributions showing 75% uncertainty bounds with *P*-values comparing the median exponential root profile parameter (*K*_ROOT_) values for each tree species within each stand. (c, d) Boxplots of calibrated exponential root profile parameter (*K*_ROOT_) values for each tree species at each site and the *P*-value comparing *K*_ROOT_ values for maple trees at Site A and Site B.

The white spruce RWU distribution determined from calibration was significantly shallower than both ash (*P*-value < 0.001) and maple (*P*-value < 0.001) in Site A (Figure 3a). The maple tree RWU distribution was not significantly different from ash (*P*-value = 0.951) (Figure 3a). In contrast, in Site B maple trees employed the shallowest RWU distributions which were significantly different from both birches (*P*-value < 0.001) and oaks (*P*-value < 0.001) (Figure 3b). In Site B, birch and oak RWU depths were not significantly different from each other (*P*-value = 0.619) (Figure 3b). Comparison of maples across Sites A and B indicated that maple trees in Site A exhibited significantly deeper RWU than maples in Site B (*P*-value < 0.001) (Figure 3c and d).

### Drought impacts on transpiration and transpiration water ages

**Figure 4:**
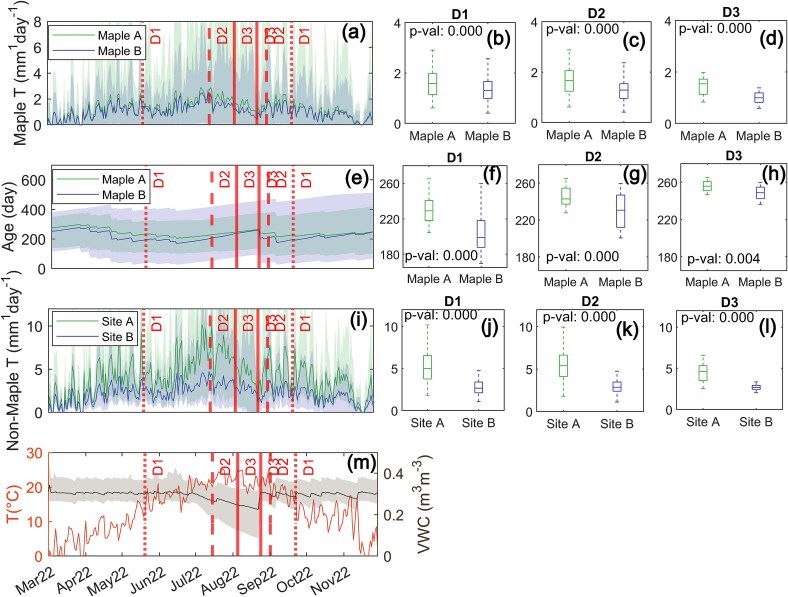
(a) Simulated daily transpiration of maple trees at Site A and Site B, (e) simulated age of water taken up by maple trees at Site A and Site B, (i) simulated daily transpiration of tree species besides maple at Site A and Site B. (m) Mean daily temperature recorded at the NCDC Storrs, Connecticut station and simulated volumetric water content (VWC) in the top 20 cm of the soil depth profile. Red lines represent the temporal duration of each USDM drought severity designation D1–D3. (b–d) Mann–Whitney *U*-test analyses of median simulated transpiration of maple trees at Site A and Site B, (f–h) median simulated water age of maple trees at Site A and Site B, (j–l) median simulated transpiration of tree species besides maple at Site A and Site B during each USDM drought severity.

Across the 2022 growing seasons (March–October), a D1 to D3 classification drought evolved, exposing trees to regionally low shallow soil moisture availability and high atmospheric water demand (Figure 4m). Across all drought severities, model simulations suggested that the deeper rooting maples in Site A exhibited higher model-simulated daily transpiration than maple trees in Site B (*P*-values < 0.001; Figure 4b–d). The model-simulated age of water taken up by maple trees at Site A was significantly older than by maples in Site B (*P*-values < 0.005). Simulated daily transpiration by all neighboring trees (excluding maple) at Site A was higher than Site B for drought classifications D1 (*P*-value < 0.001), D2 (*P*-value < 0.001) and D3 (*P*-value < 0.001).

**Figure 5:**
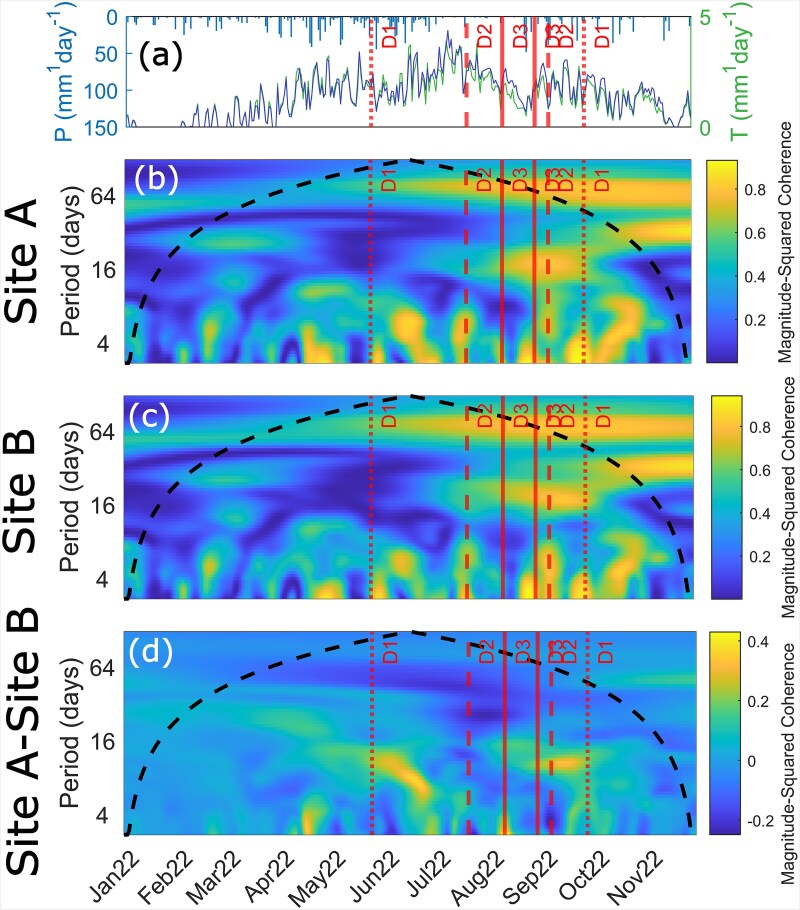
(a) Daily precipitation recorded at the NCDC Storrs, Connecticut station and simulated transpiration in maple trees at Site A and Site B showing drought severity designations D1–D3. (b, c) Wavelet coherence analysis between the time signals of simulated precipitation and transpiration of maple trees at Site A and Site B, respectively. (d) The difference between Site A and Site B precipitation and simulated maple tree transpiration coherence.

Wavelet coherence analysis between daily precipitation and simulated daily transpiration rates by maples at Sites A and B showed that simulated maple transpiration at both sites was consistently positively correlated with precipitation at a period of less than one week (i.e., maples in both sites increased simulated transpiration in response to recent rainfall) (Figure 5a–c). During the period of drought, maple trees in Site A were more positively correlated with 30-day precipitation than Site B (Figure 5b–d), whereas maples in Site B were more positively correlated with 7- to 16-day precipitation totals than in Site A (Figure 5b–d).

## Discussion

### Impact of neighboring species on maple root water uptake depths and transpiration

Our estimated genus-level rooting depths agree with several previous studies showing that maple trees both rooted and relied on shallower soil moisture than neighboring oak trees (Lyford and Wilson 1964, Lyford 1980, Matheny et al. 2017). Observed differences in neighboring clade RWU depths further supports previous findings of species functional hydraulic strategies resulting in variable responses to similar resource availabilities (Ford et al. 2011*a*, 2011*b*, Thomsen et al. 2013, Matheny et al. 2014). Evidence for variations in red maple RWU depths determined by neighboring tree species composition carries significant implications for our understanding of water cycling in mixed-species forested ecosystems. It has been posited that vegetation rooting depths can be explained using simple phylogenetic relationships (Swenson 2014, Knighton et al. 2021) or as a function of the local hydrologic conditions (Fan et al. 2019). The results of this study suggest a greater complexity where tree species interactions may drive rooting depths (Cabal et al. 2020, Agee et al. 2021). Numerically describing this level of complexity in hydrologic- and earth systems-models would require revisiting plant functional types as a framework for modeling of plant–water interactions (Anderegg et al. 2022, Jimenez-Rodriguez et al. 2024) and further study on the drivers of interspecific competition and growth within the root zone.

Our observed samples indicated that the majority of root mass in both stands existed in the upper 50 cm (Figure S1 available as Supplementary data at *Tree Physiology* Online). However, rooting depth estimates derived from process-based model calibration to water volume and isotopic data (Figure 3) suggested deeper water uptake than would be inferred from measured root mass profiles in soil cores. This potential disagreement may imply that water uptake depths in these stands are not proportional to root mass (Bachofen et al. 2024) or that a substantial portion of water transpired by individual trees with shallow roots is water that is redistributed from deeper to shallower soils (Montaldo and Oren 2022). Another possible explanation is that model calibration may only be capable of discriminating between water uptake between 0 and 30 cm and soils > 30 cm due to soil moisture isotopic homogenization at depth (Sprenger et al. 2016), which we do observe in some months (Figure S3 available as Supplementary data at *Tree Physiology* Online). Finally, substantial root mass may exist below 1 m that was not observed via auguring due to the higher gravel and rock content of the subsurface or horizontal heterogeneity in root density.

### Drought impacts on transpiration and transpiration water ages

Our findings suggest that multiple water uptake depth strategies were utilized by neighboring trees within each of the two mixed-species stands, matching the conclusions of prior studies (Gebauer et al. 2012, Chitra-Tarak et al. 2018, Brum et al. 2019, Cabal et al. 2020, Agee et al. 2021, Knighton et al. 2021, Wang and Callaway 2021, Nehemy et al. 2022) and dissimilar to other mixed-species stands that showed evidence of competition for soil moisture in the upper 50 cm despite available water in deeper layers (Gaines et al. 2016, Magh et al. 2020). While species identity (Knighton et al. 2024) and local hydrologic conditions (Fan et al. 2017, Charlet de Sauvage et al. 2024) likely influence RWU depths, our study highlights that some observed RWU variation is likely attributable to stand-level tree species compositions (Figures 1 and 3) adding to the conclusions of parallel research (Silvertown et al. 2015, Brum et al. 2019, Grossiord 2020).

Variable RWU depths in mixed-species stands may reflect complementary water use strategies to support stand-level transpiration during reductions of accessible water (Brum et al. 2019, Knighton et al. 2020*b*). Avoiding direct competition for a given water pool may allow all soil water layers to remain more hydrologically stable given that the total water demand by all plants is partitioned more evenly across soil layers. This mechanistic explanation supports the hypothesis of niche partitioning of subsurface water (Grossiord 2020, Rog et al. 2021, Wu et al. 2022).

Our modeling suggested that RWU depths were slightly lower in Site A than B (Figure 3a and b) and that non-maple transpiration for Site A was significantly higher than that of Site B throughout the period of drought (Figure 4i–l). Our findings further support that differing hydraulic strategies drive divergent transpiration and growth response to water limitation and stress within mixed stands (Matheny et al. 2014, Chitra-Tarak et al. 2018). For instance, the higher simulated transpiration of non-maple trees in Site A compared with Site B (Figure 4i–l) may be attributable to niche partitioning of water sources in non-maple clades at Site A (Knighton et al. 2020*b*), whereas non-maple trees at Site B likely compete at similar depths (Figure 3). We note that variations in simulated transpiration rates across the stands were likely attributable to other mechanisms beyond rooting depths including differences in stomatal conductance (*gs*_MAX_, Figures S4 and S5 available as Supplementary data at *Tree Physiology* Online) (Ford et al. 2011*a*, Matheny et al. 2014), sensitivity to VPDs (*gs-vpd*, Figures S4 and S5 available as Supplementary data at *Tree Physiology* Online) (Ford et al. 2011*a*) or soil matric potentials (*λ*_BC_, Figures S6 and S7 available as Supplementary data at *Tree Physiology* Online).

Deeper roots can provide a connection to older and more stable subsurface water sources that are replenished on seasonal timescales (Brinkmann et al. 2018, Allen et al. 2019, Luo et al. 2023). Deep-rooting trees may be able to conserve water resources during drought by accessing greater water availability in deep soils, and are therefore less likely to become water-stressed than shallow rooting trees (Doussan et al. 2006, Niinemets 2010, He et al. 2013, Brum et al. 2017, 2019). However, studies carried out over longer timescales have shown that long-duration droughts can disproportionately affect transpiration and mortality of deeper-rooted tree species when water in deeper soil layers becomes depleted (Mueller et al. 2005, Chitra-Tarak et al. 2018, Gutierrez Lopez et al. 2021, Knighton and Berghuijs 2023). For example, Mas et al. (2024) observed decreased transpiration rates of select species in mixed stands during severe drought conditions in a Mediterranean ecosystem despite water source partitioning, highlighting that soil water volumes will ultimately impose a limit mitigation on drought stress mitigation via heterogeneous RWU depths, particularly in mixed stands in arid ecosystems. Our study was conducted in an energy-limited region (annual potential evapotranspiration < mean annual precipitation), receiving a mean annual precipitation of 1410 mm^1^year^−1^. Therefore, root zone water was often not the limiting resource for plant transpiration. It is therefore possible that stand-level organization of tree rooting depths is not the primary determinant of mean annual transpiration rates.

Prior studies have shown substantial variations in transpiration (Granier et al. 1996, Wullschleger et al. 2001, Ewers et al. 2002, Baldocchi 2005, Knighton et al. 2020*a*) and drought tolerance (Forrester and Bauhus 2016, Pardos et al. 2021, Knighton and Berghuijs 2023) across mixed-species forested stands. Despite similar taxonomic diversity, soils and climate across the two stands, we estimated significantly different responses to drought across the two stands (Figure 1). Our ecohydrological modeling serves as a hypothesis for ecosystem functioning with a mechanistic basis (i.e., conservation of mass and energy). This foundation may help to explain some of the variation observed in prior studies. Changes in forest drought tolerance are likely the result of complex interplay between atmospheric change, changes to water movement within catchments, and the hydraulic strategies and positions of the individual trees. Limitations of plant transpiration therefore may be best defined from the perspective of evapotranspiration water ages which represent the composite impact of plant traits, the timing and availability of water arriving within the root zone, and atmospheric plant water demand (Maxwell and Condon 2016, Miguez-Macho and Fan 2021, Hahm et al. 2022, Knighton and Berghuijs 2023).

### Limitations and extensions

Paired-site designs are commonly used to study vegetation-water interactions (Andréassian et al. 2012, Som et al. 2012, Michalet et al. 2024) but individual paired studies are, by definition, low sample size experiments. Our study was carried out in two nearby plots with similar climate, soils and topography, but varied tree species compositions. Despite low sample sizes, interpreting results in the context of ecohydrological models for change detection can make the conclusions of such experiments more robust (Knighton et al. 2020*a*, Smith et al. 2020). However, we stress that these findings should be tested in other ecosystems with different climate, soils and species compositions to determine if changes in RWU depth with neighboring species composition is limited to red maples in the northeastern US, or if this behavior occurs in other settings.

Measurement of stable isotopes in the environment is a challenging task (von Freyberg et al. 2022) with documented uncertainties and potential sources of bias extending from sample collection, storage, transport, water extraction techniques and analysis (Millar et al. 2022). We collected observations in triplicate to quantify environmental heterogeneity and followed best practices established at the time of the experiment, though we acknowledge that the most appropriate methods for field data collection, storage, processing, laboratory analysis and data interpretation are evolving, and that new techniques may yield new insights.

Finally, we analyzed all collected data within the context of an ecohydrological model which helpfully eliminates several statistical assumptions about empirical data (Li and Knighton 2023), but also introduces new uncertainty in the forms of meteorological forcing data, model structure and parameter estimation (Porporato et al. 2015, Kuppel et al. 2018*b*, Li et al. 2023*a*) and the need to find agreement across parallel streams of imperfect field data. For instance, EcH_2_O-iso assumes that RWU is a non-fractionating process, in line with the majority of studies; however, several have found evidence of the contrary exemplified by certain species and in certain ecosystems (Ellsworth and Williams 2007, Barbeta et al. 2020). Further, EcH_2_O-iso does not account for hydraulic redistribution, which likely introduces uncertainty in simulating stand-level water fluxes (Hafner et al. 2021). The calibration algorithm that we employed (Ala-aho et al. 2017) identifies a suitable subset of a large number of simulations with random parameter values based on the model fit to all environmental observational datasets (Figure 2). We note that fitting an imperfect model to imperfect data resulted in some tradeoffs across the calibration space, where some observations were not reproduced perfectly by the model. This resulted in wide uncertainty bounds for some simulated stable variables (Figure 2) though this was accounted for in all statistical tests. Some of this uncertainty could possibly be reduced through data collection using techniques for in-situ measurement of soil and xylem water δ^18^O (Seeger and Weiler 2021, Kühnhammer et al. 2022, Landgraf et al. 2022). More complex model representations of plant water stress (Verhoef and Egea 2014, Kennedy et al. 2019, Simeone et al. 2019, Li et al. 2021) may also help to improve model fit to empirical measurements and support more robust conclusions in future studies.

## Conclusions

Understanding species-level RWU depth variations in mixed stands is key to answering questions of forest hydrologic regulation and resilience to external perturbations. Our study aimed to determine if within-stand species composition was associated with RWU strategies of individual red maple trees, and if variable red maple tree rooting strategies resulted in differences in transpired water by maples and neighboring tree species. We provided evidence that plant RWU depths of red maple trees varied significantly with the composition of neighboring species. Further, we observed significant differences in the volume and ages of water transpired by red maple trees and neighboring species, providing evidence of variable hydrometeorological exposure resulting from heterogenous RWU strategies. Our research highlights RWU depth as a key factor for observed variations in mixed-species transpiration. This study may provide further grounds to explore variations in mixed-species stand transpiration and resilience across different climate zones and stand species compositions. The findings of this research carry significant implications for the development of ecohydrologic and earth systems models.

## Supplementary Material

Supplemental_011425_tpaf049

## Data Availability

All soil and plant measurements collected in Sites A and B are publicly available in the following repository: https://www.hydroshare.org/resource/8996065d3ba34907a018be9b4369c1d3/.
