# Supplementary material for: Red maple tree root water uptake depths are influenced by neighboring tree species composition
Source: Tree Physiol. 2025 Apr 23;45(5):tpaf049. doi: 10.1093/treephys/tpaf049 (PMC12100743; doi:10.1093/treephys/tpaf049)
Supplement: Supplemental_011425_tpaf049 [file supplemental_011425_tpaf049.docx]

Red Maple Tree Root Water Uptake Depths are Influenced by Neighboring Tree Species Composition: Supplemental Information

Matthew Sobota^1^, Kevin Li^2^, James Knighton^3^

1.2.3. Department of Natural Resources and the Environment, University of Connecticut, Storrs, Connecticut, USA

**9. Supplemental**


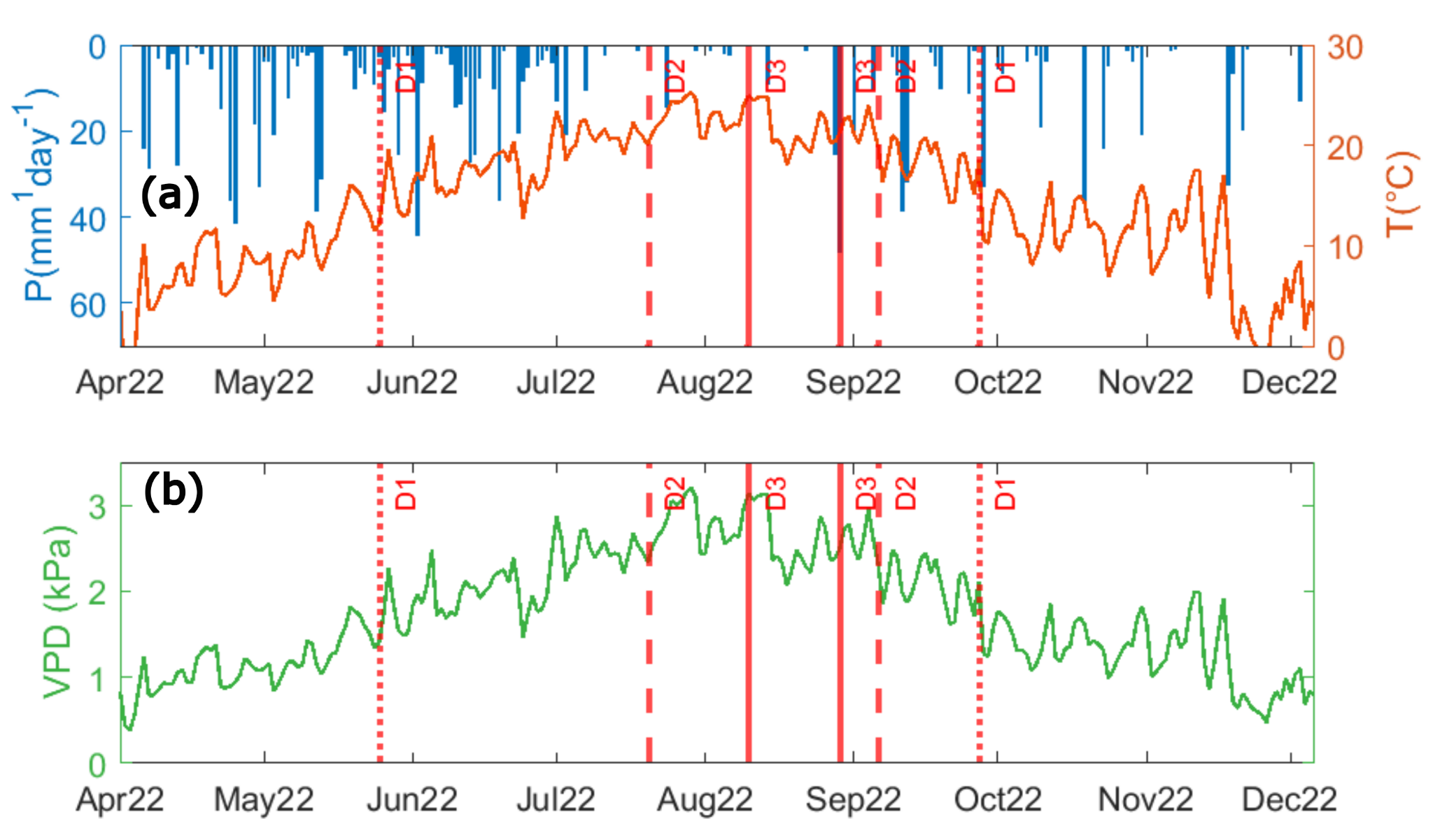


*Figure S1. Meteorological conditions at Site A and Site B during the summer 2022 drought severities designations D1 - D3. a) Daily precipitation (blue) and mean daily temperature (orange) recorded at the NCDC Storrs, Connecticut station. b) Daily vapor pressure deficit (green) derived from daily NCDC temperature and daily gridMET relative humidity at Site A and Site B.*


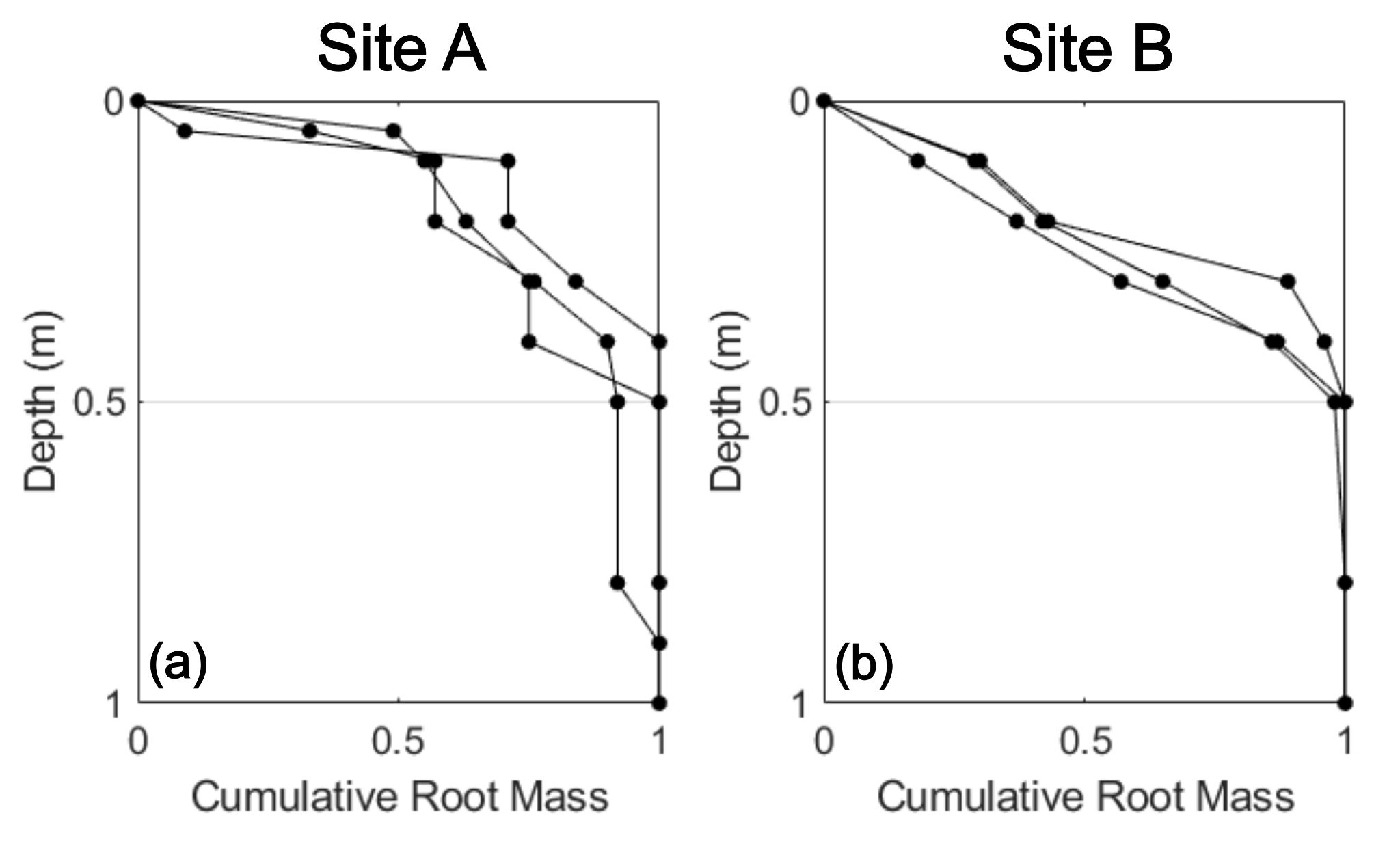


*Figure S2. Measured root mass depth profiles in Sites A and B.*

*
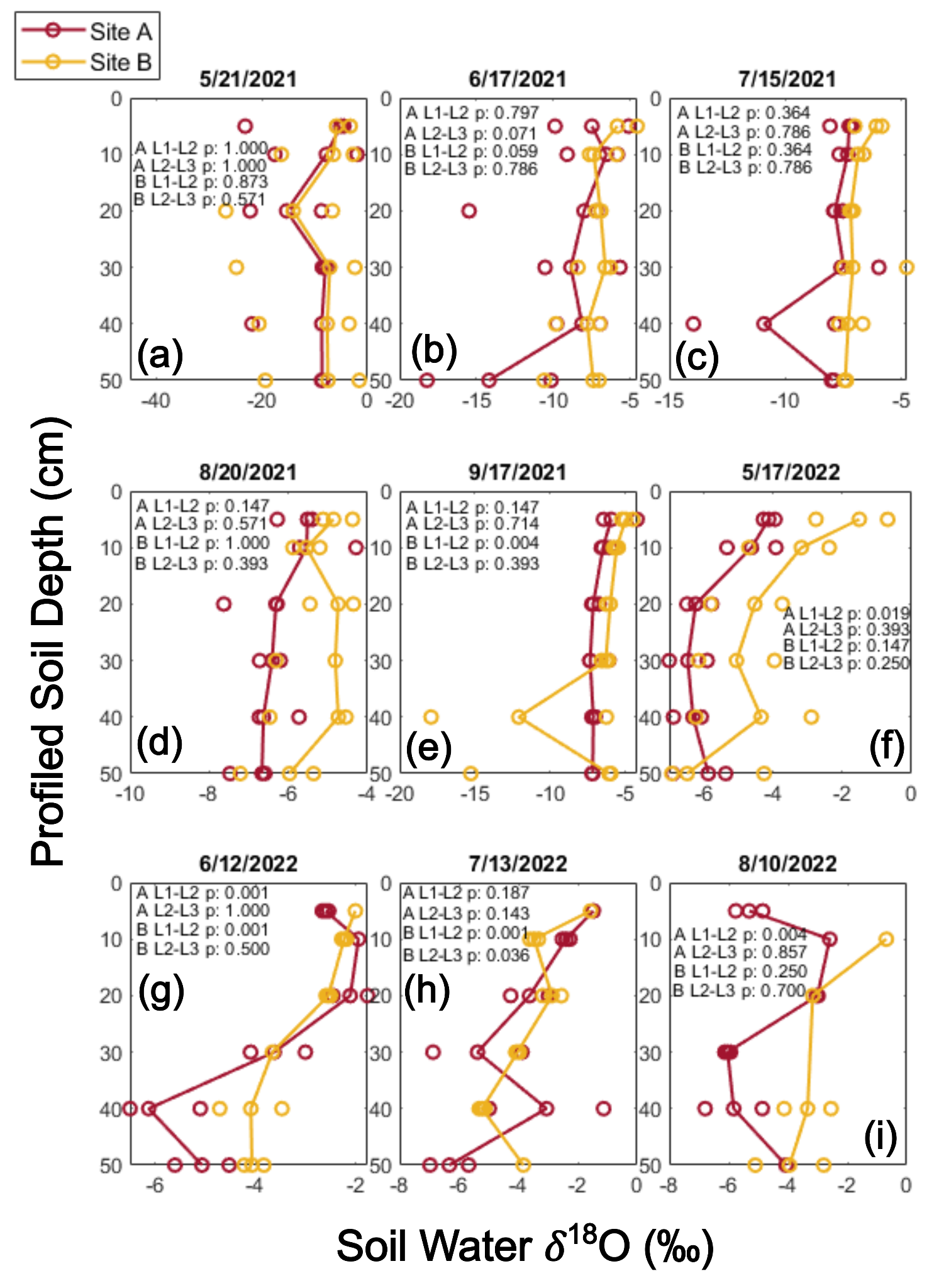
*

*Figure S3. Observed soil water* 𝛿*^18^O compositions on each sample date at Site A (red) and Site B (orange). Connected points represent the daily median* 𝛿*^18^O compositions at each measured depth. Daily minimum and maximum* 𝛿*^18^O values are also included for each sampled depth. We include Mann-Whitney U-test analyses of the observed soil water* 𝛿*^18^O compositions, comparing the* 𝛿*^18^O compositions of the 3 discretized depth layers in the model (see Section 2.3).*

|  | | | | |
| --- | --- | --- | --- | --- |
| *Table S1. EcH2O-iso Model Soil Parameters and Feasible Ranges Considered in Calibration* | | | | |
| Parameter | Description | Min | Max | Units |
| Φ | Porosity | 0.3 | 0.7 | m^3^m^-3^ |
| *α* | Albedo of bare soil | 0.1 | 0.4 | - |
| *snow* | Snowmelt coefficient | 1.0E-10 | 1.0E-3 | m^1^K^-1^ |
| *ψ*_AE_ | Soil air entry pressure | 0.1 | 0.8 | m |
| *λ*_BC_ | Brooks-Corey lambda | 2 | 12 | - |
| *KH*_SAT_ | Saturated horizontal conductivity | 1.0E-5 | 0.015 | m^1^ s^-1^ |
| Ѱ_TDP_ | Two domain pore transition tension | 0.1 | 150 | m |

|  | | | | |
| --- | --- | --- | --- | --- |
| *Table S2. EcH2O-iso Model Plant Parameters and Feasible Ranges Considered in Calibration* | | | | |
| Parameter | Description | Min | Max | Units |
| *gs*_MAX_ | Maximum stomatal conductance | 0.001 | 0.02 | m^1^ s^-1^ |
| *T*_OPT_ | Optimal plant growth temperature | 15 | 30 | °C |
| *ψ*_D_ | Limiting matric potential for RWU | 50 | 200 | m |
| *CWS*_MAX_ | Maximum canopy storage | 0 | 0.005 | m |
| *K*_BEERS_ | Beer’s light extinction coefficient | 0.1 | 0.9 | - |
| *K*_ROOT_ | Root distribution shape parameter | -15 | 15 | m^-1^ |
| *gs-*vpd | Stomatal sensitivity to vapor pressure deficits | -9.2 | -5.5 | Pa^-1^ |
| *leaf-*α | Albedo of vegetation | 0.15 | 0.3 | - |

*
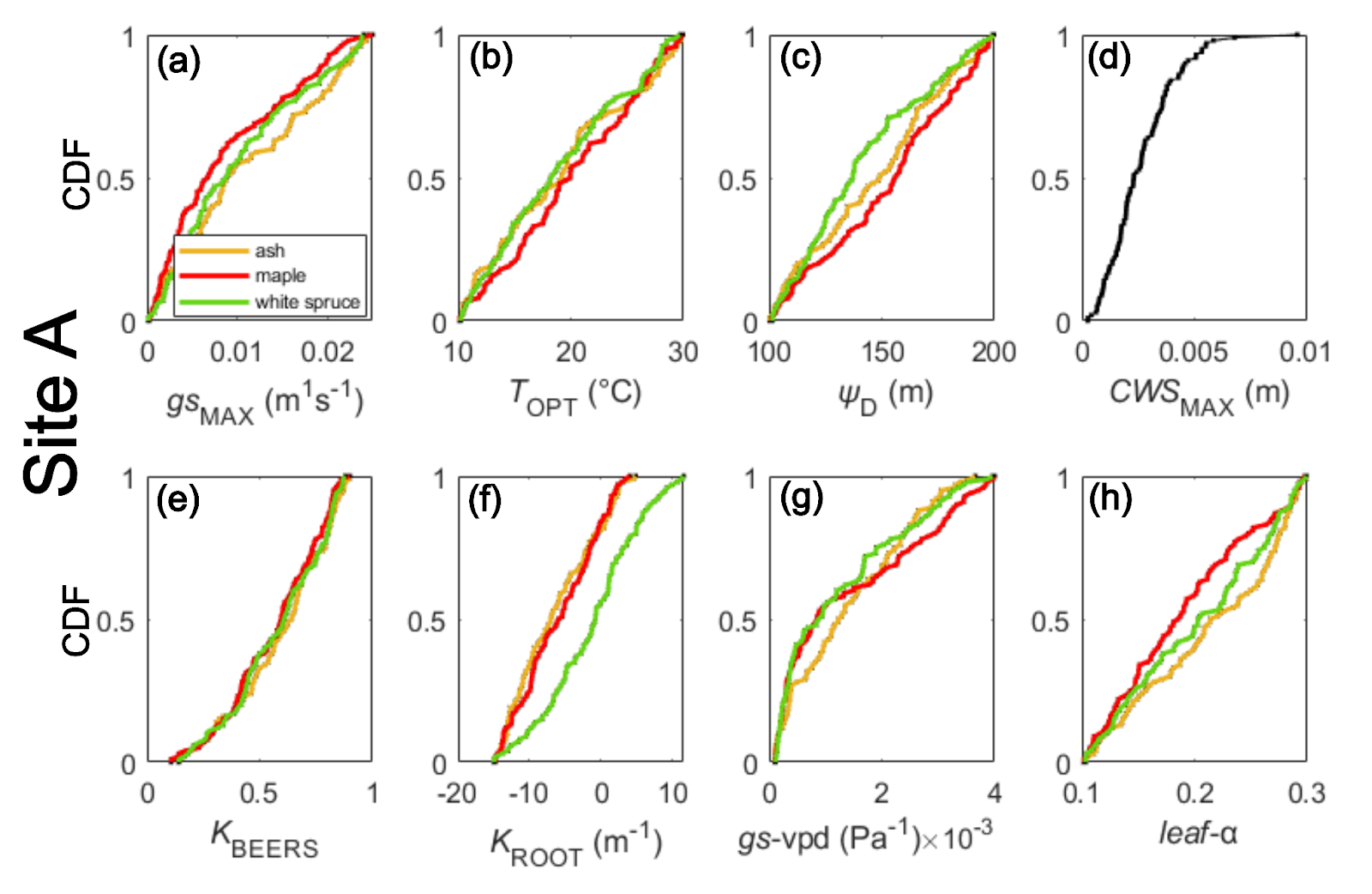
*

*Figure S4. Distribution of accepted plant parameter values (Table S2.) for each tree species after calibration at Site A. One distribution is shown for CWS_MAX_ as this parameter was not specified at the species level.*


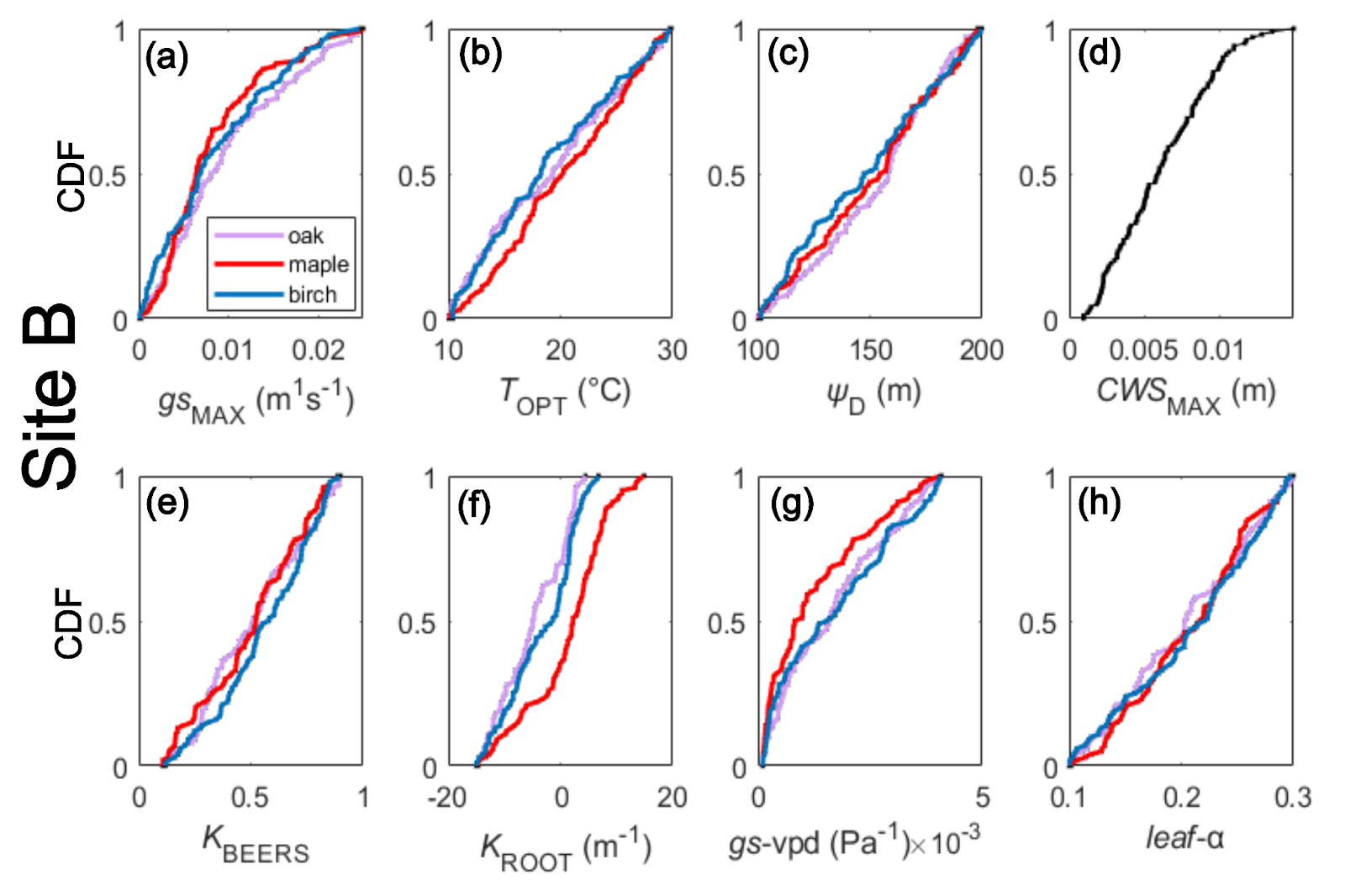
*Figure S5. Distribution of accepted plant parameter values (Table S2.) for each tree species after calibration at Site B. One distribution is shown for CWS_MAX_ as this parameter was not specified at the species level.*


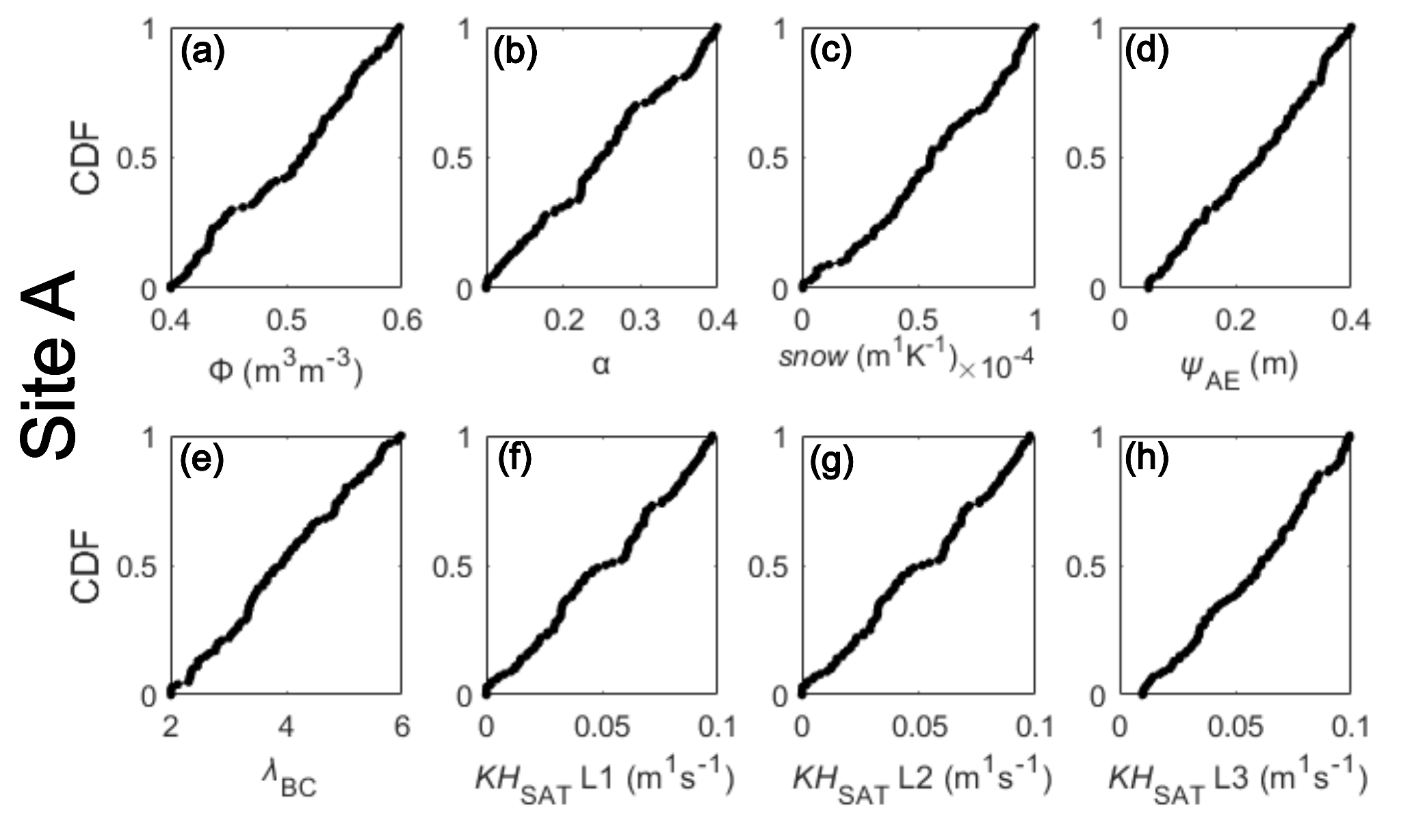


*Figure S6. Distribution of accepted soil parameter values (Table S1.) after calibration at Site A.*


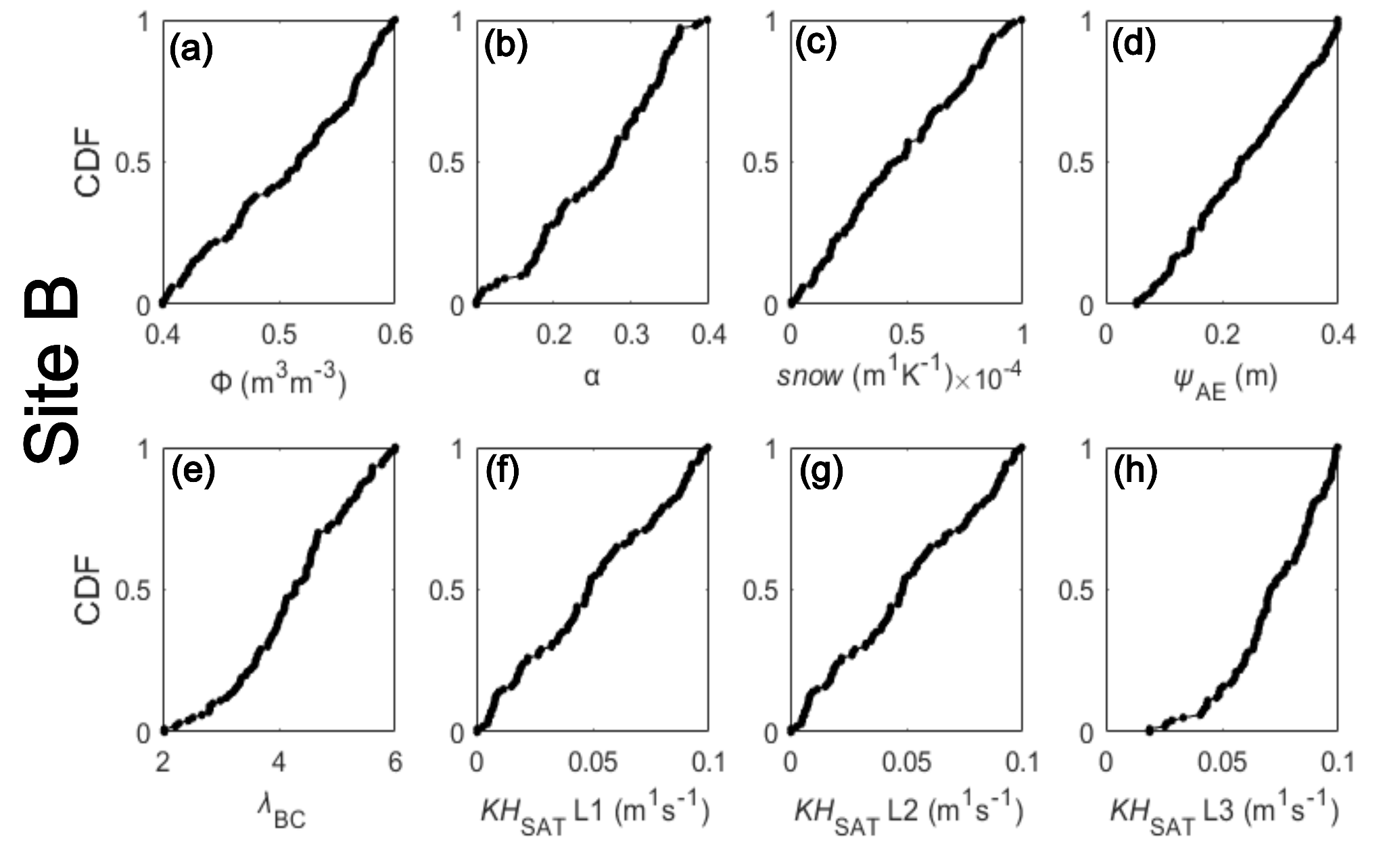


*Figure S7. Distribution of accepted soil parameter values (Table S1.) after calibration at Site B.*
